# The Vicious Cycle of Inadequate Early Detection: A Complementary Study on Barriers to Cervical Cancer Screening Among Middle-Aged and Older Women

**Published:** 2007-09-15

**Authors:** Corinne R Leach, Nancy E Schoenberg

**Affiliations:** Graduate Center for Gerontology, 306 Wethington Health Sciences Building; Department of Behavioral Science, University of Kentucky, Lexington, Kentucky

## Abstract

**Introduction:**

Although rates of invasive cervical cancer have declined precipitously over the past 50 years, nearly 10,000 new cases and 3700 deaths result from this cancer annually. Given the efficacy of early detection, invasive cervical cancer should no longer constitute a health threat; however, national studies reveal that many women, especially older women, do not receive Papanicolaou (Pap) tests.

**Methods:**

In this complementary study, we examined data from the National Health Interview Survey focusing on the correlates of screening for women aged 55 years or older, an age group in which invasive cervical cancer rates escalate and rates of obtaining Pap tests decline. To more richly understand grounded perspectives, we queried 25 women who were rarely or never screened about factors and circumstances underlying their decision not to obtain a Pap test.

**Results:**

Quantitative data indicate an association between Pap test use and demographic factors (being married, being younger, and having suburban or urban residence) and access to preventive care (obtaining mammograms, having a regular source of health care, and having contact with an obstetrician/gynecologist). Participants who provided qualitative data echoed this theme of inadequate use of preventive services, particularly among women with weak social ties, who were older, and who lived in rural areas. Shortages of health care professionals and a lack of continuity of care and privacy contribute to suboptimal prevention.

**Conclusion:**

A vicious cycle emerges: many women decline to pursue preventive care because of competing health and financial demands and insufficient resources to seek care. When such women do go to the doctor's office, they feel chastised by providers, which alienates them and thwarts future preventive care.

## Introduction

Precipitous declines (a 75% reduction over the past 5 decades) have occurred in incidence and mortality from invasive cervical cancer (ICC) because of the widespread use of the Papanicolaou (Pap) test to detect cervical abnormalities and improved technology to treat them (1,2). Despite these tremendous strides, the American Cancer Society estimates that 9700 new cases and 3700 deaths in 2006 were due to cervical cancer (3).

Although these rates are significant in themselves, two factors elevate the public health significance of cervical cancer. First, technological advances in early detection and treatment render cervical cancer a nearly preventable disease (4). Women who receive an early (stage I) ICC diagnosis have a 5-year survival rate of 92.4%, compared with a 16.5% 5-year survival rate among women with advanced ICC (5). Second, despite the successes associated with early detection and treatment, certain groups of women — including those from Appalachia, those with lower incomes, and those from older age groups — remain at disproportionate risk for cervical cancer. However, trends are encouraging. In 1970, 68% of women had had a recent (within the previous 3 years) Pap test; by 1997, this percentage was nearly 80%, and in 2000, about 84% (6).

Significant predictors of Pap test use involve resource issues, including low income (6–8); lack of a usual source of health care (7,9); lack of regularity in seeing health professionals (7,10-12), including general practitioners (10,12) and obstetricians/gynecologists (OB/GYNs) (11,12); and nonuse of other preventive services (13). Studies focusing on sociodemographic patterns of Pap testing suggest that low education levels (8,14) and being unmarried (7,8,14) decrease the likelihood of receiving a Pap test. Patterns for ethnicity have been less consistent (7,10,15,16). Living in an urban area sometimes is associated with an increased likelihood of having a recent Pap test (13), but other studies find no significant association between residence and Pap test recency (6).

Most studies suggest that younger women are more likely than older women to receive Pap tests on a regular basis (6,8,14,17). One study demonstrated that cervical cancer screening rates are significantly lower for women aged 50 to 69 years (32% not up-to-date) compared with women in younger age groups, including those aged 30 to 49 (20.3% not up-to-date) (14). As a result, older women are more likely to have regional and distant cervical cancer diagnosed than are younger women (54% versus 26%, respectively). Not surprisingly, a linear relationship exists between age and cervical cancer mortality (18).

A report on cervical cancer mortality by the National Cancer Institute identified Appalachian women as a disparity population (19): the ICC incidence rate for the United States (1995–1999) was 9.0 per 100,000 (5) compared with 13.9 in West Virginia and 15.0 in Appalachian Kentucky (20). We maintain that the challenges to obtaining Pap tests faced by women across the United States are brought into sharper relief in the Appalachian context. Barriers that thwart preventive care elsewhere — lack of health insurance, shortages of health care professionals, difficulties with transportation, and other environmental factors — are extreme challenges for women in these rural areas (21).

## Methods

We used two samples, one quantitative and the other qualitative, to examine Pap test use among women aged 55 or older. No studies have examined predictors of Pap test use specifically among this age group, and few published works have reported both survey trends and grounded, contextualized insights.

Quantitative data were taken from the 2000 National Health Interview Survey (NHIS) and focus on Pap test use within the past 3 years for women aged 55 or older without a history of hysterectomy. Swan and colleagues (6) conducted the most similar research to our study by examining this data set for predictors of Pap test use. Being unmarried, having no usual source of medical care, having no contact with a primary care provider in the past year, having a low family income, and not graduating from high school were associated with not having received a Pap test in the past 3 years. We focused on middle-aged and older women — since Pap test use drops significantly after age 50 (6,14) — and included additional variables, such as ethnicity and visits to an OB/GYN in the past year.

To capture insiders' perspectives on why women do not obtain Pap tests, we collected qualitative data throughout 2005 from a community-based sample of middle-aged and older women who were rarely or never screened in one county in Appalachian Kentucky and one county in West Virginia. The two counties have characteristics similar to other central Appalachian counties, including population size, economic standing, and health care and social service resources.

### Quantitative data analysis

We used data from the 2000 NHIS, Cancer Control Module (CCM) (22), for our analysis. The NHIS contains health information obtained from noninstitutionalized, civilian U.S. households with a maximum of one adult and one child. The CCM is part of the adult sample level of the NHIS. In 2003, the American Cancer Society modified its cervical cancer screening recommendations (23). At the time we developed this manuscript, the 2005 NHIS data were not yet available. However, since the 2003 cervical screening guidelines were not fully implemented at the time of the 2005 NHIS data collection, we do not anticipate extensive differences in the results. Of the 38,633 households sampled in the entire 2000 NHIS, 72.1% (32,374) of adults aged 18 or older responded to the CCM questionnaire. Our sample consists of women aged 55 or older without a history of hysterectomy (N = 3301).

The dependent variable was dichotomous: individuals either had or had not had a Pap test in the past 36 months. American Cancer Society recommendations for cancer screening at the time of the interview included at least one Pap test every 3 years for women 18 years of age or older with a cervix (24,25). The analysis included the following potential predictors: 1) age, 2) education level, 3) income, 4) marital status, 5) number of mammograms in the past 6 years, 6) talking with or visiting a health professional in the past 12 months, 7) talking with or visiting an OB/GYN in past 12 months, 8) having a usual source of preventive or routine care, 9) residence in a metropolitan statistical area (MSA) or non-MSA area, and 10) race/ethnicity.

The NHIS used a complex stratified multistage probability design that was age-adjusted and that oversampled African Americans and Hispanics using the 2000 census population. Data were analyzed using descriptive analysis and logistic regression in SPSS (SPSS Inc, Chicago, Illinois) and SAS 9.1 (SAS Institute Inc, Cary, North Carolina) to adjust for the complex sampling and using person weights to adjust for census-level data on sex, age, and race/ethnicity. Using data from women who provided complete information on all variables studied, we assessed the relationship between receiving a Pap test within the past 3 years and the women's demographics, whether they had received other cancer screening (e.g., mammograms), and whether they had visited a health professional or OB/GYN within the past year.

### Qualitative data analysis

The central Appalachia region, comprising mainly West Virginia, Kentucky, Ohio, and Tennessee, shares a cultural, economic, and resource base and has disproportionately high rates of cervical cancer (19). To better understand this cervical cancer burden, we studied two central Appalachian counties with comparable population sizes, economic structures, and health care capacity. The Kentucky county had 42,421 people, of which 30% lived below the federal poverty guideline and 39% had less than a high school education. The West Virginia county had 37,710 people, of which 24% lived below the federal poverty guideline and 63% had less than a high school education.

To assess what rarely or never-screened middle-aged or older central Appalachian women consider determinants of Pap test use, two local, trained middle-aged women conducted in-depth interviews consisting of structured and semistructured questions. These women were community and social workers who have lived all of their lives in the Appalachian communities in which they conducted the interviews and had extensive experience interviewing Appalachian residents. To familiarize the interviewers with research, we had them participate in extensive training sessions in which they were taught about cancer prevention, cervical cancer, and Pap tests.

The interviewers recruited the women for the study through snowball sampling (26) from a community college cafeteria, a low-income housing project, and a senior citizens center. Since identifying rarely or never-screened women is a sensitive and difficult task, we relied on the trustworthiness and reputations of the local interviewers who would discreetly ask women from these locations several screening questions. Screening included questions about age and residence (if not known) and questions about disease prevention, including when they last had a doctor's checkup and when they last had a mammogram and Pap test. Inclusion criteria were rarely (3 or more years since last Pap test) or never receiving screening for cervical cancer, residing in central Appalachia, and being middle aged or older (55 or older). Of women who were eligible and who were asked by the interviewer to participate, approximately 20% refused to do so.

Consistent with standard qualitative sampling, including theoretical saturation, participants were recruited and interviewed and data were analyzed simultaneously (27). On completion of 23 interviews, no new data emerged, and we considered our data collection complete (interviews with two additional women already had been scheduled and served as further verification).

For women who were interested in participating in the survey, an appointment was arranged for an interview, generally at the participant's home. The interviewers explained the project, administered informed consent documents, and conducted semistructured interviews. Interview guides were shaped by ecological theories of behavior that focus on the individual, social networks, the provider and health care system, and the community environment (28). All procedures were approved by the University of Kentucky's Institutional Review Board. At the end of the session, participants were provided with a $25 honorarium and information about accessing screening services, including the National Breast and Cervical Cancer Early Detection Program, with which most were unfamiliar.

The tape-recorded sessions were transcribed for subsequent analysis. Two trained researchers independently did line-by-line or axial coding, in which a label or code is affixed to chunks of text (29). To ensure rigorous, systematic, and comparable analysis, we compiled the codes and themes into a codebook. To enhance verification, two researchers coded identical text portions, establishing an intercoder reliability rating of .85 or greater (30). Researchers also met to discuss concerns or discrepancies.

## Results

### Quantitative results

We screened NHIS data for appropriateness of multivariate analysis. Analyses detected no multicollinearity among predictor variables, including variables with highly skewed distribution (having a usual source of health care and having seen a health professional in the past year). For the final analyses, we excluded 1078 cases because of missing data. The only apparent differences between the initial sample (N = 3301) and the final sample (N = 2223) were that the original sample had a lower percentage of women reporting receipt of a Pap test in the past 3 years (78.6% compared with 84.7% in the final sample) and a higher percentage of women having seen an OB/GYN in the past year (72.5% compared with 66.6% in the final sample). Descriptive statistics for the final sample are presented in Table 1.

The variables used in a logistic regression analysis to predict whether someone received a Pap test within the past 3 years were being Hispanic, non-Hispanic black, or non-Hispanic other; marital status; income; residing in an MSA; categorical age; education level; number of mammograms in the past 6 years; having seen a health professional in the past year; having seen an OB/GYN in the past year; and having a usual source of health care. As shown in Table 2, being married, living in a metropolitan area, having seen an OB/GYN in the past year, having a usual source of health care, being younger, and having a higher number of mammograms in the past 6 years were associated with a higher likelihood of receiving a Pap test within the past 3 years. Race/ethnicity, income, and education level did not significantly contribute to the final model.

### Qualitative Results

**Sample**

The 25 participants, two-thirds of whom were white and one-third of whom were black, ranged in age from 55 to 79, with a median age of 62. Educational attainment was limited, with 42% receiving 12 years of education or fewer. Annual household incomes were similarly modest; 22% had incomes of less than $5000, 35% had incomes of $5000 to $14,999, 25% had incomes of $15,000 to $24,999, and 18% had incomes of $25,000 or higher. Two women had private health insurance, five lacked health insurance, and 18 (72%) mentioned Medicare, Medicaid, or dual eligibility as their primary source of health insurance. The median number of years since their last Pap test was 8, with a range of 3 years to never for having been screened.

**Determinants of cervical cancer screening**

In-depth interviews revealed extensive convergence with the NHIS data. Participants described how older age, living in a geographically isolated place, and limited social ties converge to thwart Pap testing. A younger participant noted:

There's a lot of ladies, mostly older ladies, who live way up in the hollers (hollows) who just would never come down here and get tested. They's way too old-fashioned, or embarrassed or something. Or they got too much to do and no one is looking out for them because they might be widowed or something. I personally know a lot of ladies like that.

Discussing growing older and more isolated, another woman echoed this perspective:

I ain't exactly like that, but . . . I can see what you mean. You're in the house all alone, no one is getting after you about your health. And then, really . . . you got these aches and pains and the last thing you are really thinking about is getting that horrible test, especially when you haven't been personal with a man for a while. Why do I need that test? I gotta say, it don't make much sense for me to try to get a ride to the doctor's for this test that I probably have to pay for out of pocket when I really don't think anything is wrong.

Being older conferred numerous challenges to Pap test receipt, including competing attention from more pressing health conditions and having more limited incomes. Being unmarried, as was the case for approximately one-third of this sample, added to these challenges by limiting health vigilance or access (i.e., no one pressuring you to take care of yourself or transporting you to an appointment) and by perceived insufficient need to undergo a gynecological check-up because of limited sexual activity.

Living in a geographically remote environment further compounded the challenges of being screened, particularly for a health activity given low priority. Women discussed numerous tangible community challenges (i.e., bad roads, inadequate transportation, lack of health care professionals). A 65-year-old woman who did not drive discussed the lack of public transportation services:

You know, out here, there's no bus or taxi you can call up and have them take you over to the doctor. If you're like me, then you have to plan all of the things you have to do real carefully and go when you can . . . when someone can take you. [But] you can't just drop in and get a check-up, so you can make an appointment, but who really knows if you'll get your ride then.

Participants also described health care systems barriers to getting a Pap test. Negative opinions of preventive health services seem to stem from several sources: insufficient and inconsistent availability of health care professionals, inadequate health insurance, lack of confidentiality with medical care, and negative attitudes toward formal health care.

The scarcity of health care professionals in rural and underserved communities leads to a constellation of associated problems, as noted among our participants and in the literature (31). Participants described too few doctors (leading to inadequate hours for clinics, a dire shortage of specialists, few female physicians, and physicians who are viewed as "good doctors" being overworked), too little privacy (one woman reported that the test results of a very sensitive problem were known at her worksite before she even returned to her job that afternoon), and constant turnover of health care professionals, especially among international medical graduates and residents.

Many women seldom make appointments with physicians unless they are in great discomfort or believe their health to be in danger, suggesting that the doctor is a last resort when experiencing pain or disability. As described by the participant below, who had not "been to a doctor's office except to get something for bronchitis trouble I had, and that was about 5 years ago," this lack of care tends to become habitual:

You got your people who are really looking after themselves. They don't smoke, they eat right, you know. And these are the same people who pop over to their doctors on a regular basis and say, "Here I am — ain't I healthy or what?" Then there's the rest of us (*laughs)*. We're a mess and we never do go. And we should be the first ones there! But it's sort of a vicious cycle — you don't ever see a doctor, but you probably should, so you really don't want to go unless you absolutely have to.

Participants voiced concern about how health care providers chastise them for not seeking regular preventive care, which only made them reluctant to seek care, both to prevent health problems and to treat emerging problems. One woman explained how a nurse yelled at her for not having regular blood pressure checks:

I was coming in about my sugar diabetes and she told me that I was about to kill myself with high blood pressure and that if I didn't follow up [on my appointments], I wouldn't live to see my granddaughter graduate [from high school]. I'm not sure about that, but I didn't like getting threatened.

Another woman noted that she seldom visited a health care provider,

. . . when I do go, it's usually for something that is real bad, like that time when I had to get my eyes checked. That scared me, just thinking about losing my eyesight. But things that don't trouble me . . . no, I don't really bother. To tell the truth, I'm more troubled by the thought of those doctors yelling at me because I don't always do what they say than I am worried about each ache and pain.

Others described the possible humiliation they might face during such an examination, particularly being smokers or overweight, behaviors they acknowledged as problematic and likely to meet with disapproval from health care professionals. One woman who last had a Pap test 14 years ago noted,

I hate to think about getting up on the doctor's table, with this fat bottom of mine. Lord knows what the doctor and maybe even the nurse would say. I know that I'd be better off if I wasn't so big, and I can only think they'd keep letting me know that I needed to lose some weight. I can't stand the thought of it.

Participants' narratives defy a simple conclusion that the women lack an appreciation for prevention or lack transportation. Instead, tangible barriers to prevention combined with a historical and community context that may relegate prevention to low-priority status thwart Pap testing.

## Discussion

The percentage of participants who reported having had a Pap test within the American Cancer Society guidelines was similar to that found among women aged 25 or older (6). However, because data from the survey were self-reported, many women who did not respond to this question may not have been current with their Pap test, and some of those who did respond may have overestimated the recency of their test. Because middle-aged or older women are at heightened risk for cervical cancer, anything less than universal screening for eligible middle-aged or older women constitutes an unacceptable risk for cervical cancer. Although 84.7% seems like a large majority of women, this percentage falls far short of the *Healthy People 2010* goal of 97% of women aged 18 or older receiving a Pap test within the past 3 years (32).

Some critics might argue that a situation in which one in six women does not receive a Pap test does not appear to be a crisis, but these women tend to be the most vulnerable to poor health outcomes according to their demographics and health care use. Those women who are rarely or never screened for cervical cancer appear to be underusers of the medical care system in general. Our findings suggest that outreach efforts must be made to these reluctant users of health services, including creating a comfortable, nonjudgmental medical environment.

Two primary factors appeared to play a role in the cervical cancer screening practices among women in the quantitative and qualitative aspects of this study: demographic variables and access to and use of preventive care.

### Demographics

Our quantitative and qualitative findings suggest that being unmarried or living alone, living in an isolated place, and being older tend to decrease Pap test use. Married women were 43% more likely to be current on their Pap tests compared with all other women, similar to findings by Hewitt et al (7). Narratives from rarely or never-screened women help explicate this finding: unmarried women may not have anyone to encourage them to seek health care and might also experience greater logistical barriers, including a lack of transportation (especially salient for women who do not drive) and a lack of health insurance. Some women also discussed their perceived lack of need for Pap tests due to their current lack of sexual activity, an incorrect perception that may heighten their risk of cervical cancer.

Women from MSAs were 52% more likely to have received a Pap test in the past 3 years than were women from rural areas, a finding that is both refuted (6) and corroborated (13) by previous research. Our in-depth interviews point to several explanatory factors, including structural limitations (e.g., insufficient health care services, lack of health insurance).

With each additional 10-year age band among women aged 55 or older, the likelihood of having regular screenings decreased by 35%, a finding consistent with previous research (7,8,17). Interviews revealed that advanced age places a greater burden on women to manage other, seemingly more important health concerns ("aches and pains" along with serious chronic diseases), in addition to factors such as social and geographical isolation and greater financial constraints. These results are not consistent with a previous study on colorectal cancer screening in Appalachia in which older age was associated with a higher likelihood of having a recent screening (33). However, colorectal cancer and cervical cancer are very different conditions with different ages of screening initiation. In addition, colonoscopy is now covered by Medicare, removing a major financial barrier for older adults.

### Access to care and use of preventive care

Both the qualitative and quantitative portions of this study indicate that a key determinant of receiving a Pap test is undergoing other types of preventive screening. Although intuitive, this finding has only received a small amount of attention in the literature (13). Lyttle and Stadelman (34) found similar results, with women who received regular mammograms being more likely to receive regular colorectal cancer screenings. The qualitative interviews in our study suggested that competing health, social, and economic concerns converged with histories of self-reliance, traditional health practices (e.g., complementary and alternative medicine), and unconventional health beliefs that fall outside of formal medical encounters. Participants, many of whom have multiple, demanding, and costly chronic conditions, discussed the need to prioritize their use of time and economic and social resources. Preventive health screenings may be relegated to a secondary concern, not out of ignorance, fatalism, or lack of motivation — assumptions frequently leveled at traditionally underserved populations — but because of self-care traditions and prioritization (31). Previous research confirms that those self-care practices established earlier in life tend to remain in people's health repertoire, particularly when they continue to operate in resource-scarce conditions (35). The women in our study, and most likely many women who do not receive Pap tests, have developed health strategies consistent with their culture and circumstances (poverty and shortages of health care professionals meant mother was the doctor) and with their health priorities (managing multiple chronic conditions, including pain and some disability).

Having contact with an OB/GYN was the strongest predictor of Pap use in this sample of women, increasing the likelihood of being current by nearly eight and a half times and corroborating others' results (7,11). As suggested by our qualitative participants, those individuals who "pop over to their doctors on a regular basis and say, 'Here I am — ain't I healthy or what?' " not only have a preventive health orientation but also have the means to get to providers' offices and to overcome a lack of health care professionals and an inadequate continuity of care. Participants do not mistrust or refute the legitimacy of the medical establishment; however, factors such as lower socioeconomic status and traditions of self-reliance may encourage women to give low priority to prevention (31,36).

Race/ethnicity, income, and education level did not significantly predict current Pap test screening. However, Hewitt and colleagues (7) suggest that individuals aged 25 or older with less than a high school education and with a lower income are less likely to receive regular screenings. Additionally, Swan and colleagues (6) found that higher income and higher levels of education are associated with receiving regular Pap tests among women 25 and older. These results suggest that distinct factors may play a larger role in screening uptake among middle-aged and older women.

### Limitations

One limitation of self-report surveys such as the NHIS is that many women overreport Pap test use (37). Another limitation is that many women in the sample did not respond to one of the variables in the study, which may influence the generalizability of the quantitative results. A limitation of our quantitative analyses was that two variables (having a usual source of health care and having seen a health professional in the past year) had little variability, perhaps explaining why seeing a health professional in the past year did not significantly predict Pap testing.

Finally, while the predictive and explanatory powers of this study were enhanced by complementary methods, the data came from separate data sets. We cannot necessarily explain the decisions of the women in the quantitative portion of this study through the narratives of the qualitative participants. However, qualitative insights help to explain patterns observable through the quantitative data, while the quantitative data allow us to examine associations among variables with confidence in their reliability and generalizability. We cannot definitively state that the factors and circumstances influential for the 25 women interviewed are generalizable to other locales in Appalachia or in the rural United States.

### Conclusion

Our results suggest that a vicious cycle pertains to underscreening of vulnerable populations. Older, unmarried women living in rural areas who do not have a usual source of preventive or routine health care, who do not visit or speak with an OB/GYN each year, and who do not receive other preventive care such as mammograms are less likely than other women to receive regular Pap tests. Rarely or never-screened women explain this pattern in terms of logistical challenges (e.g., not having adequate transportation), perceptual barriers (e.g., worry that health care professionals will chastise them for being overweight), and lack of social support (e.g., a supportive person who is concerned about the woman's health). When they do overcome these barriers, women report feeling stigmatized in the medical encounter, which undermines their use of preventive services.

The best predictor of screening is the recommendation of a health care professional (38–40). In the absence of supportive family and friends, health professionals must provide encouragement in addition to consistent and clear recommendations to patients of all ages, with special attention to middle-aged or older women. Kind words and sensitivity to the environmental and personal constraints faced by many traditionally underserved women would most likely facilitate screening. In addition, expanded support should be provided for programs such as the National Breast and Cervical Cancer Early Detection Program, which assist lower-income and rarely or never-screened women in receiving needed cancer prevention services.

## Figures and Tables

**Table 1:** Characteristics of Women Aged 55 Years or Older Without a History of Hysterectomy (Final Sample N = 2223) in Two Appalachian Counties, National Health Interview Survey, 2000

Characteristic	No. of Respondents (%)
**Had Pap test in past 3 years**	
Yes	1882 (84.7)
No	341 (15.3)
**Age, y**	
55-64	877 (39.5)
65-74	702 (31.6)
75-84	526 (23.7)
≥85	118 (5.3)
**Marital status**	
Currently married	880 (39.6)
Not currently married	1343 (60.4)
**Race/ethnicity**	
Hispanic	222 (10.0)
Non-Hispanic black	265 (11.9)
Non-Hispanic white	1691 (76.1)
Non-Hispanic other	45 (2.0)
**Education level**	
No education	13 (0.6)
1st-8th grade	267 (12.0)
9th grade	61 (2.7)
10th grade	90 (4.0)
11th grade	141 (6.3)
High school graduate/ General Educational Development certificate	793 (35.7)
Some college	345 (15.5)
Associate's degree	159 (7.2)
Bachelor's degree	216 (9.7)
Master's degree	106 (4.8)
Higher degree	32 (1.4)
**Annual household income, $**	
<20,000	895 (40.3)
≥20,000	1328 (59.7)
**No. of mammograms in past 6 years**	
0	132 (5.9)
1-2	482 (21.7)
3-4	403 (18.1)
≥5	1206 (54.3)
**Saw a health professional in past year**	
Yes	2125 (95.6)
No	98 (4.4)
**Saw an OB/GYN in past year**	
Yes	742 (33.4)
No	1481 (66.6)
**Residing in an MSA**	
Yes	1756 (79.0)
No	467 (21.0)
**Have a usual source of health care**	
Yes	2157 (97.0)
No	66 (3.0)

Pap test indicates Papanicolaou test; OB/GYN, obstetrician/gynecologist; MSA, metropolitan statistical area.

**Table 2:** Logistic Regression Model Predicting Whether or Not Women (N = 2223) Aged 55 Years or Older Had Received a Current Pap Test in Two Central Appalachian Counties, National Health Interview Survey, 2000

Predictor Variable	Odds Ratio	Wald *Χ* ^2^	*P* Value
Married	1.43	4.10	.04
Income	0.86	0.74	.39
Reside in an MSA	1.52	5.78	.02
Saw a health professional in past year	1.33	0.82	.36
Saw an OB/GYN in past year	8.45	61.49	<.001
Have a usual source of health care	3.07	8.14	.004
Hispanic	1.60	1.94	.16
Non-Hispanic black	1.25	0.56	.45
Non-Hispanic other	0.46	2.48	.12
Categorical age	0.65	29.98	<.001
No. of mammograms in past 6 years	1.71	151.99	<.001
Education level	1.03	1.41	.23

Pap test indicates Papanicolaou test; MSA, metropolitan statistical area; OB/GYN, obstetrician/gynecologist.
